# Extracellular RNA transfer from non‐malignant human cholangiocytes can promote cholangiocarcinoma growth

**DOI:** 10.1002/2211-5463.13294

**Published:** 2021-09-22

**Authors:** Yu Ota, Kenji Takahashi, Shin Otake, Yosui Tamaki, Mitsuyoshi Okada, Irene Yan, Kazunobu Aso, Satoshi Fujii, Tushar Patel, Masakazu Haneda

**Affiliations:** ^1^ Division of Metabolism and Biosystemic Science, Gastroenterology, and Hematology/Oncology Department of Medicine Asahikawa Medical University Japan; ^2^ Departments of Transplantation and Cancer Biology Mayo Clinic Jacksonville FL USA; ^3^ Department of Laboratory Medicine Asahikawa Medical University Japan

**Keywords:** epithelial‐mesenchymal transition, extracellular vesicles, microRNA, secretome, tumor microenvironment

## Abstract

Extracellular vesicles (EV) within the cellular secretome are emerging as modulators of pathological processes involved in tumor growth through their ability to transfer donor‐derived RNA into recipient cells. While the effects of tumor and stromal cell EVs within the tumor microenvironment have been studied, less is known about the contributions of normal, nontransformed cells. We examined the impact of EVs within the cellular secretome from nonmalignant cells on transformed cell growth and behavior in cholangiocarcinoma cells. These effects were enhanced in the presence of the pro‐fibrogenic mediator TGF‐β. We identified miR‐195 as a TGF‐β responsive miRNA in normal cells that can be transferred via EV to tumor cells and regulate cell growth, invasion, and migration. The effects of miR‐195 involve modulation of the epithelial–mesenchymal transition through direct effects on the transcription factor Snail. These studies provide *in vitro* and *in vivo* evidence for the impact of normal cellular secretome on transformed cell growth, show the importance of EV RNA transfer, and identify mechanisms of EV‐mediated transfer of miRNA as a contributor to tumor development, which may provide new therapeutic opportunities for targeting human cholangiocarcinoma.

## Graphical abstract

Cholangiocyte cell secretome can include extracellular vesicles (EV) containing tumor‐suppressive noncoding RNA such as miR‐195 and contribute to microenvironmental modulation of cholangiocarcinoma growth. miR‐195 can suppress the epithelial–mesenchymal transition and inhibit tumor invasion, migration, and growth.

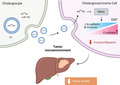

**Abbreviations**
3′UTR: 3′untranslated region
CCA: cholangiocarcinoma
CM: conditioned medium
EMT: epithelial–mesenchymal transition
EV: extracellular vesicle
FBS: fetal bovine serum
HCC: hepatocellular carcinoma
miRNA: microRNA
ncRNA: noncoding RNA
TME: tumor microenvironment
YAP: yes‐associated protein

Cholangiocarcinoma (CCA) is a primary liver epithelial cancer of the liver and is often diagnosed at an advanced stage when intrahepatic spread and metastases have occurred and which contribute in part to their poor overall prognosis [1]. The tumors often arise in the context of a dense stroma emphasizing the need for defining the contributions of a fibrogenic milieu within the tumor microenvironment (TME) and its impact on tumor growth or response to therapy. Both tumor and nontumor cells can secrete bioactive macromolecules and extracellular vesicles (EV) into the extracellular space. Collectively, these comprise the cellular secretome and are profound modulators of the TME [2].

The participation of the cellular secretome in modulating pathological processes is increasingly recognized. The beneficial effects on tissue repair and regeneration associated with mesenchymal stem cells can be mediated by the paracrine effects of EVs and other molecules within their secretome [3]. EVs are an essential component of the cell secretome, with roles in intercellular communication [4, 5, 6]. In tumor settings, EV‐mediated communication can contribute to tumor growth and metastases through the transfer of proteins or RNA [7, 8]. We have previously shown that EVs derived from hepatocellular cancer contain noncoding RNAs (ncRNAs) that can modulate signaling pathways related to resistance to chemotherapy or hypoxic stress in recipient cells [9, 10, 11]. We have also shown that CCA cell‐derived EVs can transfer miR‐30e and modulate the epithelial–mesenchymal transition (EMT) pathway in recipient CCA cells [12]. EMT has been implicated as a mechanistic contributor to tumor cell invasion and migration [13].

While the effects of tumor and stromal cell EVs within the TME have been studied, less is known about the contributions of normal, nontransformed cells. In biliary tract disorders such as primary biliary cholangitis, senescent cholangiocytes can modulate inflammation and biliary damage through alterations in their secretome [6]. However, the impact and influence of nonmalignant cells on transformed cell growth and behavior within tissues are not well understood. Therefore, we sought to test the hypothesis that nonmalignant cells within the local TME can secrete factors that target and modulate transformed cell function. Our studies provide *in vitro* and *in vivo* data on the impact of normal cellular secretome on transformed cell growth, show the importance of EV RNA transfer, and identify mechanisms of EV‐mediated transfer of miRNA as a contributor to tumor development in CCA.

## Materials and methods

### Cell lines, culture, and reagents

HuCCT1, HuH28, and OZ human CCA cell lines were obtained from the Japanese Cancer Research Resources Bank (JCRB) Cell Bank (Osaka, Japan), and TFK‐1 and RBE were purchased from the RIKEN BRC through the National Bio‐Resource Project of the MEXT (Tsukuba, Ibaraki, Japan) [14]. MMNK‐1 nonmalignant human cholangiocytes were obtained from the JCRB. HuCCT1, HuH28, TFK‐1, and RBE cells were maintained in RPMI 1640 medium (Thermo Fisher Scientific, Waltham, MA, USA) with 10% fetal bovine serum (FBS) and 1% penicillin–streptomycin (Thermo Fisher Scientific). OZ cells were cultured in Williams' medium (Thermo Fisher Scientific) containing 10% FBS and 1% penicillin–streptomycin. MMNK‐1 cells were cultured in Dulbecco's’ modified Eagle's medium (DMEM) high‐glucose medium (Thermo Fisher Scientific) with 5% FBS and 1% penicillin–streptomycin. All cells were cultured at 37 °C with 5% CO_2_. For all EV studies, EV‐depleted medium was prepared using exosome‐depleted FBS (Thermo Fisher Scientific). Transforming growth factor (TGF)‐β was obtained from EMD Millipore Corporation (Billerica, MA, USA), and the cells were treated with 10 ng·mL^−1^ TGF‐β for 72 h to induce the EMT.

### Isolation of conditioned medium and EVs

Cells (1 × 10^6^ cells) were seeded on 10‐cm dishes and cultured in 11 mL of EV‐depleted medium. After 3 days, the media was collected from 16 dishes and centrifuged at 300 ***g*** for 10 min, then at 2000 ***g*** for 20 min at 4 °C. The supernatant was filtered with a filtration system (Thermo Fisher Scientific) to remove cellular debris. This conditioned medium (CM) was used to isolate the EVs and for the *in vivo* experiments. The CM was ultracentrifuged at 10 000 ***g*** for 70 min at 4 °C to collect the EVs pellets, which were then resuspended in 10 mL phosphate‐buffered saline (PBS) and were further ultracentrifuged at 100 000 ***g*** for 70 min at 4 °C. The final pellet contained a heterogeneous population of EVs and was used to isolate EV RNA or in other *in vitro* experiments. EV morphology was observed by transmission electron microscopy. The protein content was measured using a bicinchoninic acid protein assay kit (Thermo Fisher Scientific), and CD63 expression was evaluated by western blotting.

### RNA isolation and quantitative real‐time polymerase chain reaction (qRT‐PCR)

Total RNA was extracted from cells using the miRNeasy Mini Kit (Qiagen, Valencia, CA, USA), and the RNA concentration was measured using a NanoDrop ND‐1000 (Nano‐Drop Technologies, Wilmington, DE, USA). RNA was treated with RNase‐Free DNaseⅠ (Qiagen) and reverse‐transcribed to cDNA using the iScript^®^ cDNA Synthesis Kit (Bio‐Rad Laboratories, Hercules, CA, USA). qRT‐PCR was performed using an Applied Biosystems^®^ 7300 Real Time PCR System (Applied Biosystems, Foster City, CA, USA) to detect mRNAs with SYBR Green I (SYBR Advantage qPCR Premix; Clontech Laboratories, Inc., Mountain View, CA, USA). The primers used were as follows: E‐cadherin forward: 5′‐TGCACCAACCCTCATGAGTG‐3′ and reverse: 5′‐GTCAGTATCAGCCGCTTTCAG‐3′; Snail forward: 5′‐TTCTCACTGCCATGGAATTCC‐3′ and reverse: 5′‐GCAGAGGACACAGAACCAGAAA‐3′; N‐cadherin forward: 5′‐TCGCCATCCAGACCGACCCA‐3′ and reverse: 5′‐TGAGGCGGGTGCTGAATTCCC‐3′; vimentin forward: 5′‐CCTGAACCTGAGGGAAACTAA‐3′ and reverse: 5′‐GCAGAAAGGCACTTGAAAGC‐3′; and RNU6B forward: 5′‐CTCGCTTCGGCAGCACA‐3′ and reverse: 5′‐AACGCTTCACGAATTTGCGT‐3′. Expression of miRNAs was assessed by the TaqMan human MicroRNA (miRNA) Assay Kit (Applied Biosystems) and normalized to RNU6B.

### MiRNA microarray analysis

The miRNA microarray profiling studies in the HuCCT1 human CCA cell lines and MMNK‐1 nonmalignant human cholangiocyte cell line were performed using a 3D‐Gene miRNA microarray platform (Toray Industries Inc., Tokyo, Japan). RNA isolation and microarray analysis were carried out as described previously [12]. Briefly, RNA was labeled with the 3D‐Gene miRNA labeling kit (Toray Industries Inc.) and hybridized onto a 3D‐Gene Human miRNA Oligo chip (Toray Industries Inc.). The annotation and oligonucleotide sequences of the probes conformed to the miRBase miRNA database (http://microrna.sanger.ac.uk/sequences/). After stringent washes, the fluorescent signals were scanned with the 3D‐Gene Scanner (Toray Industries Inc.) and analyzed using 3D‐Gene Extraction software (Toray Industries Inc.). The relative expression level of a given miRNA was calculated by comparing the signal intensities of the valid spots throughout the microarray experiments. The normalized data were globally normalized per array, such that the median signal intensity was adjusted to 25. The results were compared between HuCCT1 and MMNK‐1, or TGF‐β‐treated HuCCT1 and nontreated HuCCT1 cells. The data can be accessed through the NCBI GEO database under NCBI accession nos. GSE104629 and GSE121512.

### Transfection of miRNA mimic and inhibitor

Cells were transfected with 12.5 nm of the mirVana^®^ miR‐195 mimic, 25 nm of the mirVana^®^ miR‐195 inhibitor, or a negative control (Applied Biosystems) using Polyethylenimine Max (Polysciences, Warrington, PA, USA) according to the manufacturer’s instructions. After 48–72 h, the cells were collected and used for further experiments.

### Luciferase reporter assays

The Snail 3′untranslated region (3′UTR) firefly luciferase reporter vector and empty vector were purchased from OriGene Technologies (Rockville, MD, USA). Cells (2 × 10^5^ cells·well^−1^) were seeded on a six‐well plate and treated with 12.5 nm of mirVana^®^ miR‐195 or the negative control mimic. After 24 h, cells were cotransfected with 2.0 μg of Snail 3′UTR firefly luciferase reporter vector or empty vector, and 0.1 μg of Renilla luciferase reporter pRL‐SV40 (Promega, Madison, WI, USA) using Lipofectamine 2000 (Thermo Fisher Scientific). After another 24 h, luciferase activity was measured using the Dual‐Luciferase Reporter Assay (Promega). Relative firefly luciferase activity was normalized to Renilla luciferase activity.

### Protein extraction and western blotting

Total protein was extracted from cultured cells using cOmplete^®^ Lysis‐M EDTA‐free and cOmplete Mini EDTA‐free protease inhibitor cocktail tablets (Roche, Basel, Switzerland). Equivalent amounts of proteins from each sample were mixed with NuPAGE^®^ 4× LDS Sample Buffer (Life Technologies, Grand Island, NY, USA), separated by electrophoresis on NuPAGE® Novex 4–12% Bis‐Tris Gels (Life Technologies) and then transferred onto nitrocellulose membranes. The membranes were blocked with blocking buffer (TBST; 25 mm Tris/HCl pH 7.4, 125 mm NaCl, and 0.05% Tween‐20) with 5% bovine serum albumin (Sigma‐Aldrich, St. Louis, MO, USA) for 1 h and then incubated overnight at 4 °C with the indicated primary antibodies: mouse monoclonal anti‐human CD63 (1 : 200, FUJIFILM Wako Pure Chemical Corp., Osaka, Japan), rabbit monoclonal anti‐E‐cadherin (1 : 1000; Cell Signaling Technology, Danvers, MA, USA), goat polyclonal anti‐Snail (1 : 1000; Abcam, Cambridge, UK), rabbit monoclonal anti‐N‐cadherin (1 : 1000; Cell Signaling Technology), rabbit monoclonal anti‐vimentin (1 : 1000; Cell Signaling Technology), and mouse monoclonal anti‐β‐actin (1 : 1000; Santa Cruz Biotechnology, Dallas, TX, USA). The membranes were washed three times for 15 min with TBST and then incubated with the indicated secondary antibodies: ECL^®^ Anti‐Rabbit IgG HRP‐Linked Whole Antibody (1 : 20 000; GE Healthcare, Hatfield, UK) for E‐cadherin, N‐cadherin, and vimentin; ECL^®^ Anti‐Mouse IgG HRP‐Linked Whole Antibody (1 : 20 000; GE Healthcare) for CD63 and β‐actin; and ZyMax^®^ Rabbit anti‐Goat IgG (H + L) HRP Conjugate (1 : 100 000, Life Technologies) for Snail. Protein expression was visualized and quantified using the ChemiDoc XRS+ Imaging System (Bio‐Rad Laboratories). Relative protein expression was determined by probing the same membranes for β‐actin.

### Cell proliferation and viability assays

Cells (1 × 10^4^ cells·well^−1^) were seeded on 24‐well plates. Trypan blue staining was performed after 24, 48, 72, and 96 h, and the number of viable cells was expressed relative to cell counts at baseline. For the cell viability assay, cells (1 × 10^4^ cells·well^−1^) were seeded on 96‐well plates. Cell viability was assessed using CellTiter 96^®^ AQueous One Solution Assay (Promega) after 24, 48, 72, and 96 h. A background correction was made by subtracting background fluorescence from wells without cells.

### Cell invasion and migration assays

Cell invasion studies were performed using 24‐well Transwell^®^ permeable support 24‐well plates (Corning Corp., Corning, NY, USA). The Transwell plates were coated with prediluted Matrigel (5 μg·μL^−1^; BD Biosciences, Bedford, MA, USA) prior to experimentation. Cells (5 × 10^4^ cells·well^−1^) in 100 μL serum‐free medium were seeded in the upper chamber, and the lower chamber of the Transwell was filled with 600 μL of medium supplemented with 10% FBS. After a 24‐h incubation, the cells remaining on the upper surface of the Transwell chamber were removed with a cotton swab. The cells that had invaded through the Matrigel to the bottom of the insert were fixed and stained using Diff‐Quik^®^ (Sysmex, Hyogo, Japan). The number of migrated cells was counted in three random high‐power fields under a light microscope, and the relative number of cells was calculated. For the cell migration assays, cells (4 × 10^5^ cells·well^−1^) were seeded on six‐well plates. After cells were grown to 90% confluence, straight scratches were created using a sterile pipette tip. Floating cells were removed and the cultures were maintained. Images of wounds were taken immediately and after 6 and 12 h. Cell migration was assessed by measuring the wound closure provided by the cells.

### Tumor xenografts

Five‐week‐old male BALB/c‐nu/nu athymic nude mice were obtained from Charles River Laboratories Japan Inc. (Yokohama, Japan) and housed under a 12‐h light: 12‐h dark cycle with free access to food and water. The mice were anesthetized under isoflurane (1%–2% by inhalation during induction) before subcutaneous injections to either flank with HuCCT1 cells (1 × 10^6^ cells) transfected *ex vivo* by overexpressing miR‐195 or the nontargeted control and suspended in 0.5 ml BD Matrigel^TM^ Basement Membrane Matrix (BD Biosciences). Between 5 and 14 days after the subcutaneous injections, 0.5 mL of CM derived from miRNA‐overexpressing or control MMNK‐1 cells was injected daily into each xenograft tumor. Growth of tumor cell xenografts was monitored by serial measurements (in mm^3^), and volume was estimated using the following equation: tumor volume = π/6 × [minor axis]^2^ × major axis. The mice were euthanized, and the tumors were excised for RNA and protein extraction 20 days after the subcutaneous injections.

### Statistical analysis

Data are expressed as mean and standard error from at least three replicates. Comparisons between the groups were performed using two‐tailed Student’s *t*‐test or one‐way analysis of variance followed by the Bonferroni post hoc test. A *P*‐value < 0.05 was considered significant. Data were analyzed using prism 6 software (GraphPad, San Diego, CA, USA).

### Ethical approval and consent to participate

All animals received humane care, and studies were performed under Asahikawa Medical University guidelines for the use of animals with approval from the Animal Research Committee of the university.

## Results

### The cellular secretome of normal cells and TGF‐β can modulate the tumoral cell phenotype in CCA

**Fig. 1:**
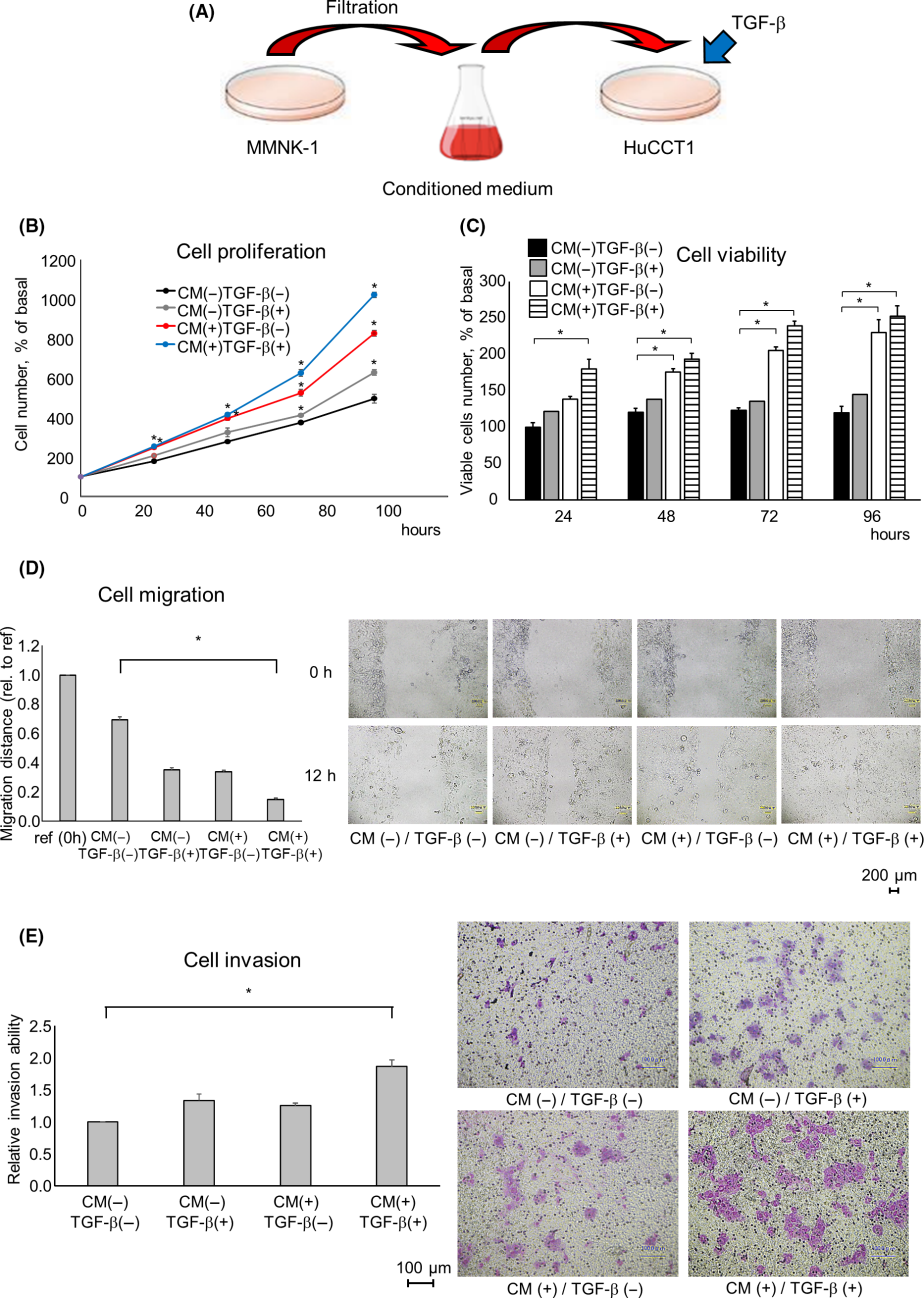
The effect of MMNK‐1 cell‐derived secretome on tumor cell phenotypes *in vitro* in the presence or absence of TGF‐β. (A) The culture medium collected from MMNK‐1 cells was filtered to remove cellular debris to prepare the CM as the cell secretome. Recipient HuCCT1 cells were incubated with or without those CM or TGF‐β. (B, C). Cell proliferation was examined using trypan blue, and cell viability was examined by the MTS assay after 24, 48, 72, and 96 h. (D) Cell migration was assessed by the wound healing assay. Wounds were made after 24 h, and the wound area was measured after 12 h. Scale bars = 200 μm. (E) Cell invasion was assessed by Transwell assay after 24 h. Scale bars = 100 μm. Data were analyzed using Bonferroni post hoc test, and bars represent mean ± SEM of three separate determinations. *P < 0.05.

To investigate the impact of nontransformed normal cholangiocytes on tumor cell phenotypes, we first optimized isolation collection of the cellular secretome as conditioned media (CM) by sequential ultracentrifugation from MMNK‐1 nonmalignant human cholangiocytes (Fig. 1A). Next, we evaluated the effects of CM on HuCCT1 CCA cells. We observed that the normal cholangiocyte secretome product could promote HuCCT1 cell proliferation and viability, as well as enhance cell migration and invasion compared to vehicle controls. CCA often arises within a dense stroma, suggesting that TME effects may occur in the presence of mediators of a fibrogenic environment such as transforming growth factor β (TGF‐β). Subsequently, we therefore examined the tumor cell responses in the presence of TGF‐β and observed a further increase in tumor cell proliferation and viability in the presence of TGF‐β (Fig. 1B,C). Similarly, TGF‐β enhanced the effects of tumor cell migration and invasion (Fig. 1D,E). These data suggested that normal, nontransformed cholangiocytes could promote tumor cell growth in the context of a profibrogenic milieu.

### miR‐195 is a component of the cholangiocyte secretome

**Fig. 2:**
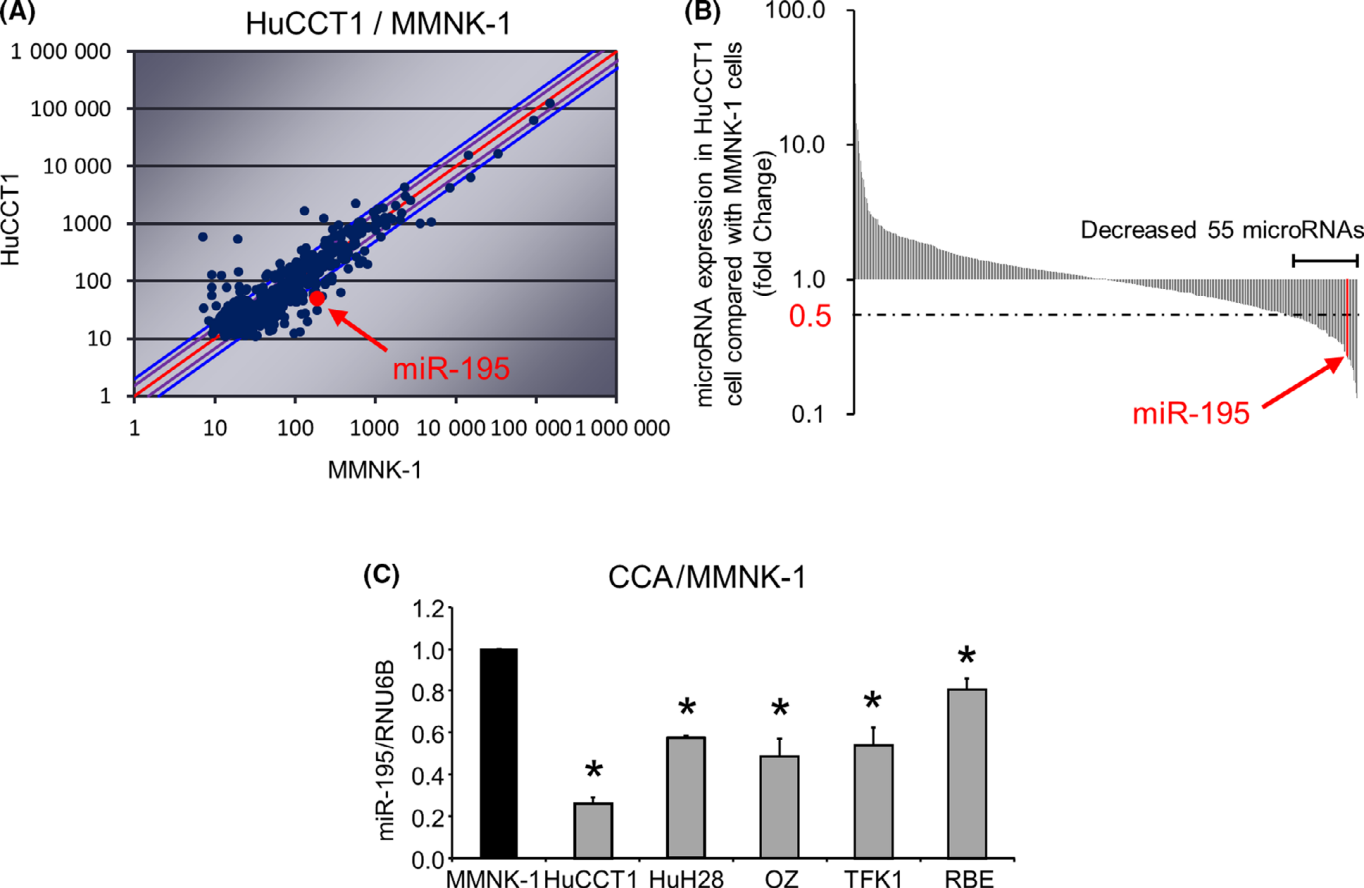
miR‐195 expression is reduced in tumor cells. (A) Expression profiling of 2555 miRNAs was performed using a miRNA microarray, in which 507 miRNAs were expressed in MMNK‐1 and HuCCT1 cells. (B) The microarray identified 55 miRNAs that were decreased < 0.5‐fold in HuCCT1 cells compared to MMNK‐1 cells. (C) RNA was isolated from human MMNK‐1 and CCA cells, and miR‐195 expression was analyzed by quantitative real‐time polymerase chain reaction (qRT‐PCR). Relative expression normalized to RNU6B is shown. Data were analyzed using Bonferroni *post hoc* test, and bars represent mean ± SEM of three separate determinations. *P < 0.05.

We next sought to evaluate the role of ncRNA within the secretome. To begin with, we analyzed global expression of 2555 miRNA in both normal and tumor cells using miRNA microarray analysis (Fig. 2A). A total of 507 miRNAs were expressed in both HuCCT1 and MMNK‐1 cells. Of these, 55 miRNAs were downregulated with < 0.5‐fold expression in HuCCT1 compared to MMNK‐1 cells (Fig. 2B). Among these, miR‐195 was one of the most downregulated miRNAs in malignant cells compared with the expression in nonmalignant MMNK‐1 cells. We next validated that expression of miR‐195 in a panel of human CCA cells and found that the basal expression level in malignant cells was 0.26–0.80 that in nonmalignant cells (Fig. 2C). The reduced expression across this panel of tumor cells suggested that miR‐195 may have tumor‐suppressive effects. Unexpectedly, incubation of HuCCT‐1 cells with CM from MMNK‐1 cells increased miR‐195 to 122% ± 6% (*P* = 0.02) compared with control media and suggested the possibility that the nonmalignant cholangiocyte secretome could have local effects on tumor cells in proximity.

**Fig. 3:**
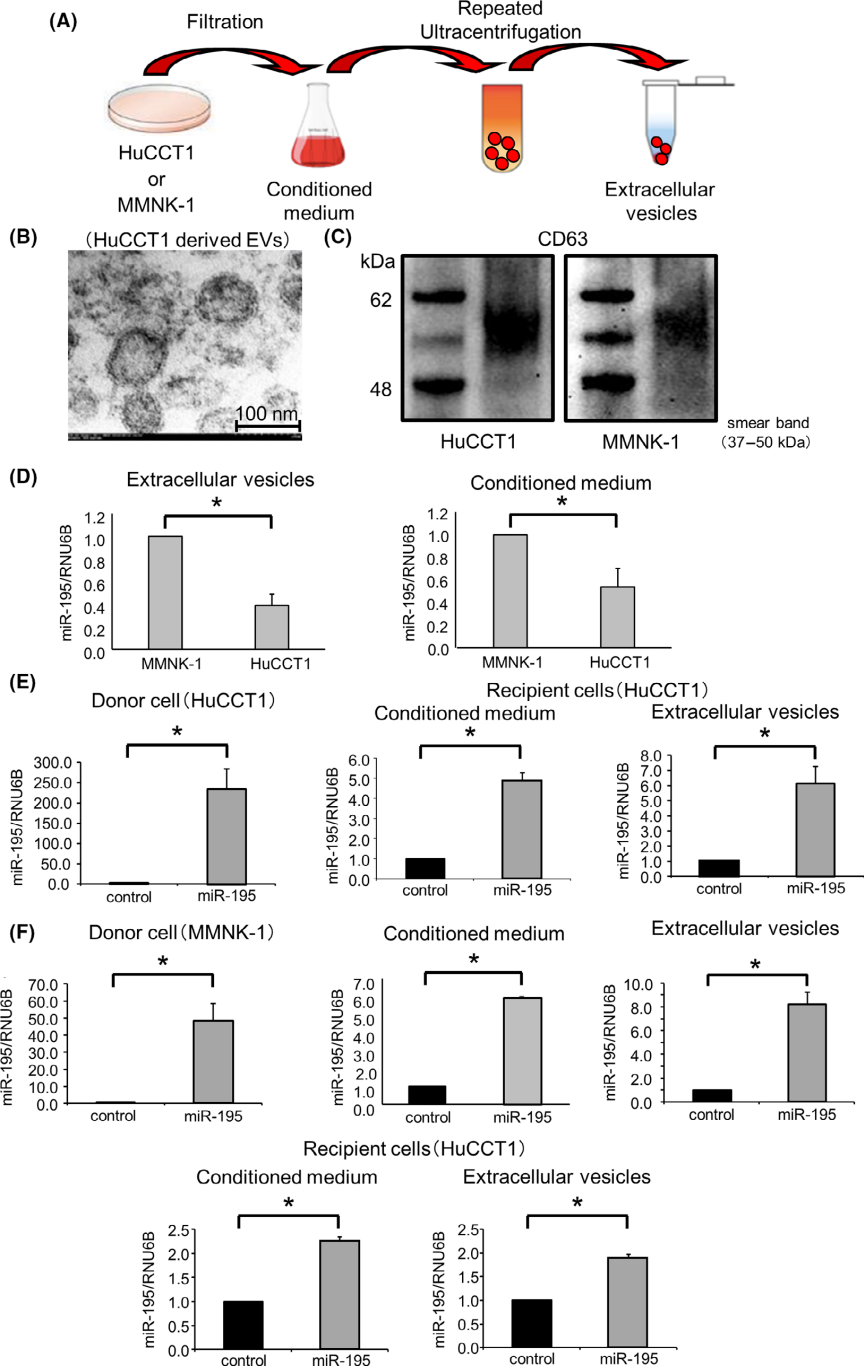
Isolation of secretome or EV enriched in miR‐195. (A) The culture medium collected from HuCCT1 or MMNK‐1 cells was filtered to remove cellular debris and used as the CM. Extracellular vesicles (EVs) were isolated from CM by repeated ultracentrifugation. (B) Electron microscopic image showing EVs isolated from HuCCT1 cells. Scale bars = 100 nm. (C) Western blotting analysis shows CD63 protein expression in EVs purified from HuCCT1 or MMNK‐1 cells. (D) Basal expression levels of miR‐195 in EVs and CM derived from MMNK‐1 or HuCCT1 cells were assessed by qRT‐PCR. (E and F) Donor HuCCT1 or MMNK‐1 cells were transfected with miR‐195 or a control mimic, and CM or EVs obtained after 72 h. CM or EVs obtained from donor HuCCT1 cells were added to recipient HuCCT1 cells. After 48 h, RNA was isolated and analyzed for miR‐195 expression in the donor or recipient cells using qRT‐PCR (E). Recipient HuCCT1 cells were incubated with CM or EVs obtained from donor MMNK‐1 cells. After 48 h, RNA was isolated and then analyzed for miR‐195 expression in the donor EVs and CM or recipient cells using qRT‐PCR (F). Data were analyzed using Student’s *t*‐test, and bars represent mean ± SEM of three separate determinations. *P < 0.05.

EV‐mediated crosstalk among tumor cells plays a key role in the TME [9]. EVs can influence the behavior of neighboring cells through the transfer of their contents such as miRNAs from donor to recipient cells. In order to evaluate the role and involvement of miR‐195 in TME cell signaling, we isolated EVs from nonmalignant MMNK‐1 cholangiocytes or HuCCT1 CCA cells. Cells were cultured in EV‐depleted medium, and EV isolated by ultracentrifugation (Fig. 3A). The isolated EVs were approximately 100 nm in size, with a lipid bilayer membrane consistent with EVs isolated from other cell types and previous reports (Fig. 3B). The isolated EVs were verified by detecting expression of CD63 (Fig. 3C).

Therefore, we examined whether miR‐195 could be transferred within EVs. miR‐195 was detected in EVs and CM derived from MMNK‐1 cells and basal expression of miR‐195 in MMNK‐1 cell EVs and CM was greater than that observed in HuCCT‐1 cell‐derived EVs and CM (Fig. 3D). The expression of miR‐195 was enforced in HuCCT1 cells by transfection with miR mimic, and the enhanced expression levels were confirmed by PCR. We observed a significant increase in miR‐195 expression in recipient HuCCT1 cells after incubation with EV that was isolated from donor miR‐195 expressing HuCCT1 cells. Similar results in recipient miR‐195 were observed with incubation with CM from miR‐195‐expressing HuCCT1 cells (Fig. 3E). We also observed a significant increase of miR‐195 expression in recipient HuCCT1 cells that were incubated with miR‐195‐enriched EVs derived from the miR‐195‐expressing MMNK‐1. Moreover, the efficiency of transfer of miR‐195 transfer within EV was very similar to that observed with CM (Fig. 3F). These data suggested that the miR‐195 present in the secretome was predominantly within EV.

### EV‐mediated miR‐195 transfer modulates the CCA cell phenotype *in vitro*

**Fig. 4:**
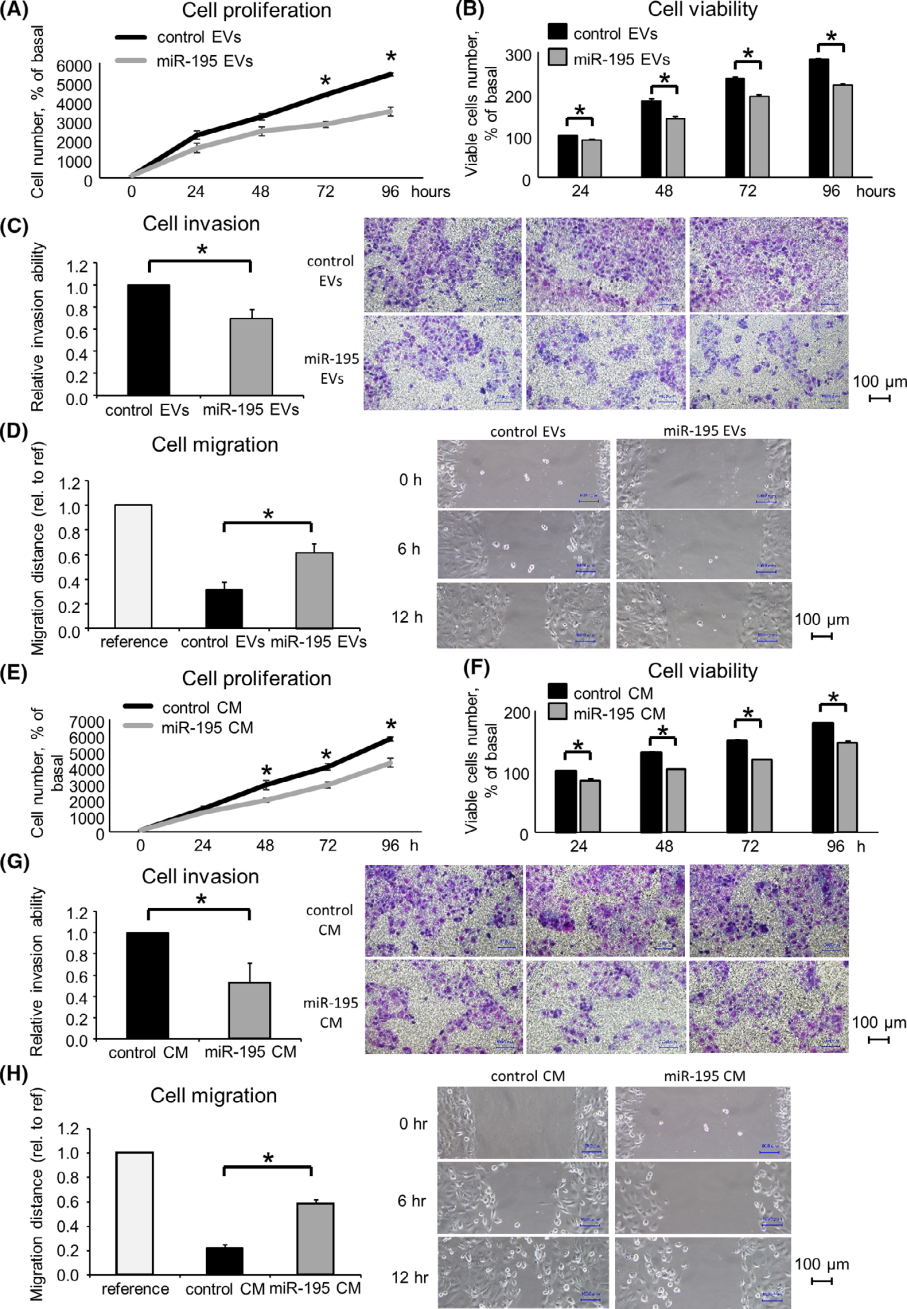
miR‐195 alters tumor cell phenotype *in vitro*. MiR‐195‐enriched EVs (A–D) or CM (E–H) or their respective controls were isolated from MMNK‐1 cells and administered to HuCCT1 cells. (A, B, E, F) Cell proliferation was examined by cell counting using trypan blue, and cell viability was examined by the MTS assay after 24, 48, 72, and 96 h. (C, G) Cell invasion was assessed by a Transwell assay after 24 h. (D, H) Cell migration was assessed by a wound healing assay. Wounds were made after 24 h, and the wound area was measured after 12 h. All scale bars = 100 μm. Data were analyzed using Student’s *t*‐test, and bars represent mean ± SEM of three separate determinations. *P < 0.05.

We next examined whether EVs isolated from nonmalignant cells could transfer miR‐195 and their impact on recipient CCA cells by examining the effects on the tumor cellular phenotype. Cell growth, invasion, and migration were analyzed in recipient cells after incubation with MMNK‐1‐derived EVs. Incubation of HuCCT1 cells with miR‐195‐enriched CM or with miR‐195 enriched EVs significantly suppressed cell proliferation, viability, invasion, and migration (Fig. 4). The EV component of CM enables the transfer of intact functional RNA across cell types without degradation. For subsequent studies on the cellular effects of miR‐195 in CCA, we used CM from nontransformed cells to not only incorporate all relevant components of the secretome but to avoid the potential impact of other diverse oncogenic cargoes studies within EV from cancer cells.

### MiR‐195 can suppress EMT pathway

**Fig. 5:**
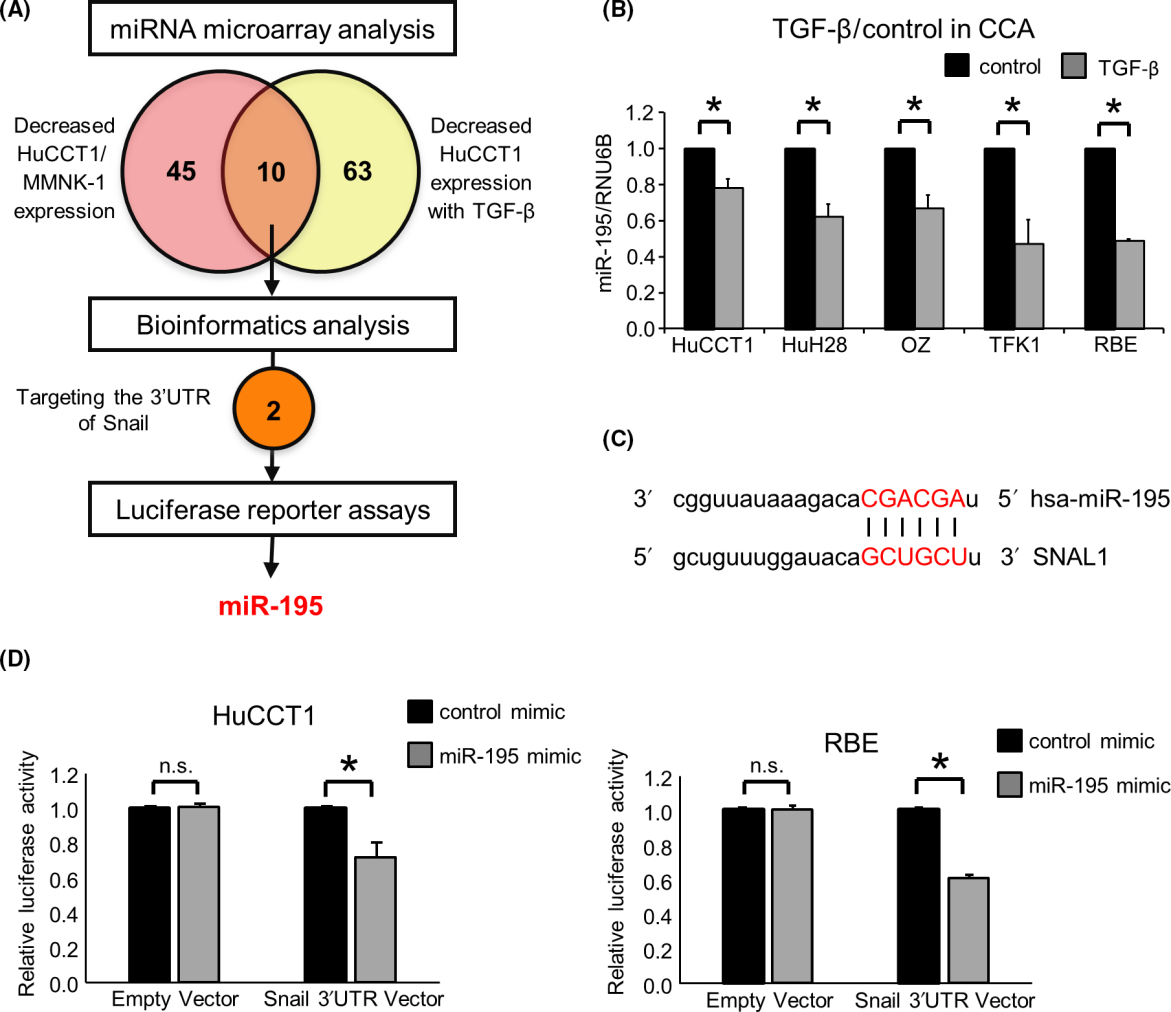
Transforming growth factor β responsive miRNA regulating EMT. (A) Schematic diagram to illustrate the process by which miR‐195 was selected. (B) CCA cells were incubated with 0 or 10 ng·mL^−1^ TGF‐β, and miR‐195 expression was assessed by qRT‐PCR. (C) miR‐195 has sequence complementarity to the Snail 3′UTR. (D) HuCCT1 and RBE cells were transfected with 12.5 nm miR‐195 or control mimic and co‐transfected with 2.0 µg of the Snail 3′UTR firefly luciferase reporter vector or empty vector, and 0.1 µg of the Renilla luciferase reporter pRL‐SV40, after 24 h. After a further 24 h, relative firefly luciferase activity was measured and normalized to Renilla activity. n. s.: not significant. Data were analyzed using Student’s *t*‐test, and bars represent mean ± SEM of three separate determinations. *P < 0.05.

**Fig. 6:**
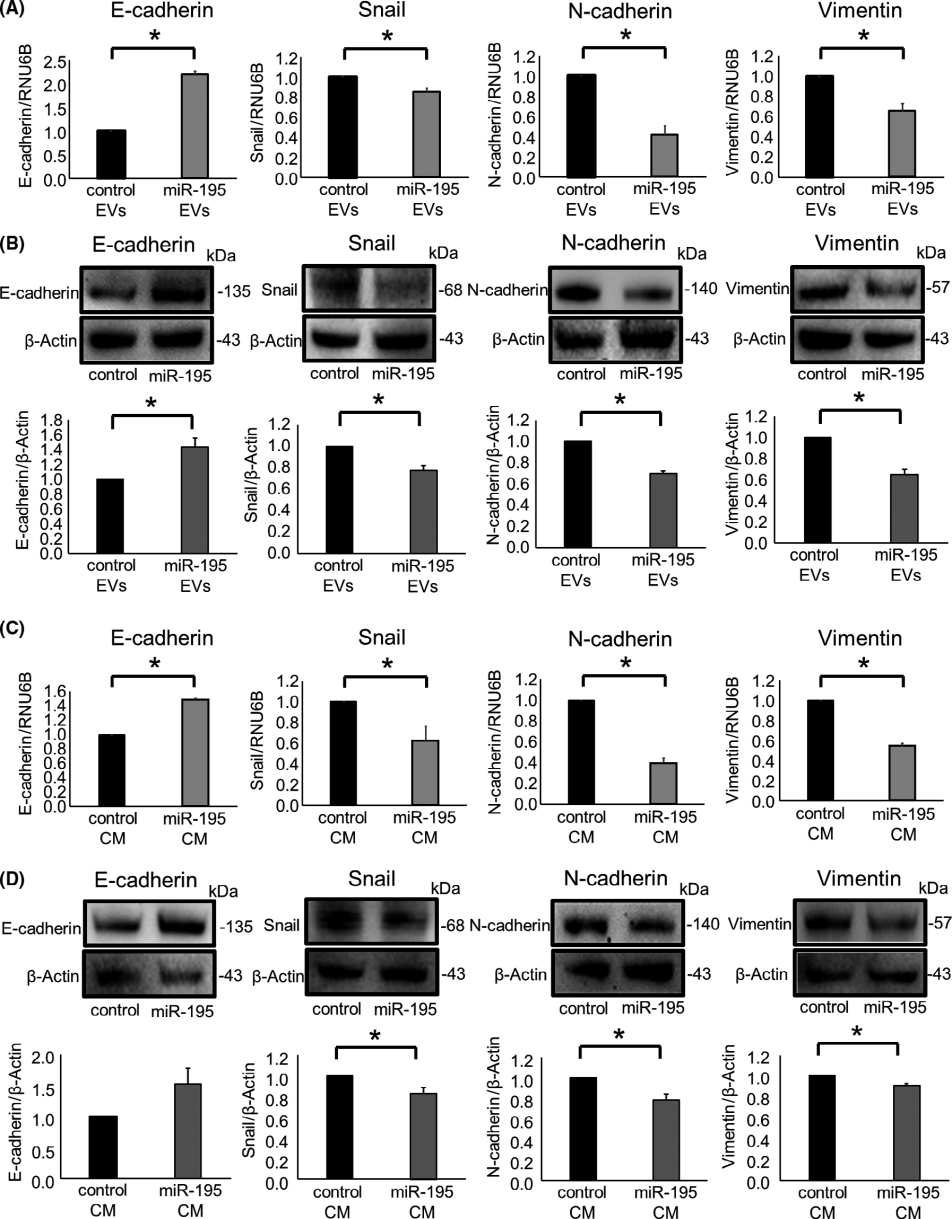
miR‐195 alters EMT‐related pathways *in vitro*. MiR‐195‐enriched EVs (A, B) or CM (C, D) or their respective controls were isolated from MMNK‐1 cells and administered to HuCCT1 cells. (A, C) After 48 h, RNA was isolated and analyzed for E‐cadherin, Snail, N‐cadherin, and vimentin by qRT‐PCR. (B, D) After 72 h, the protein was extracted and assessed for E‐cadherin, Snail, N‐cadherin, and vimentin by western blot. A representative immunoblot is shown, along with quantitative data obtained by densitometry. Data were analyzed using Student’s *t*‐test, and bars represent mean ± SEM of three separate determinations. *P < 0.05.

The EMT is an important pathway that contributes to tumor invasion and metastasis. We have previously verified that incubation with 10 ng·mL^−1^ TGF‐β induced EMT without toxicity in both HuCCT1 and RBE human CCA cell lines [12]. We evaluated the roles of miR‐195 on its effect on EMT pathway. First, we analyzed the effects of TGF‐β on miRNA expression in HuCCT1 cells (Fig. 5A). Compared with controls, 73 of 2555 miRNAs were downregulated < 0.7‐fold in HuCCT1 cells after TGF‐β treatment as described previously [12]. Because tumor‐suppressing and EMT‐suppressing miRNAs can inhibit carcinogenesis and tumor development, we focused on miRNAs with reduced expression in HuCCT1 cells and that were also further downregulated by TGF‐β. miR‐195 was among the ten miRNA identified in this manner. The expression level of miR‐195 was reduced by TGF‐β in a panel of CCA cells (Fig. 5B). Furthermore, a greater increase in E‐cadherin and a decrease in Snail, N‐cadherin, and vimentin were observed in recipient HuCCT1 cells following exposure to miR‐195 enriched EVs isolated from miR‐195‐overexpressing MMNK‐1 cells (Fig. 6A,B).

**Fig. 7:**
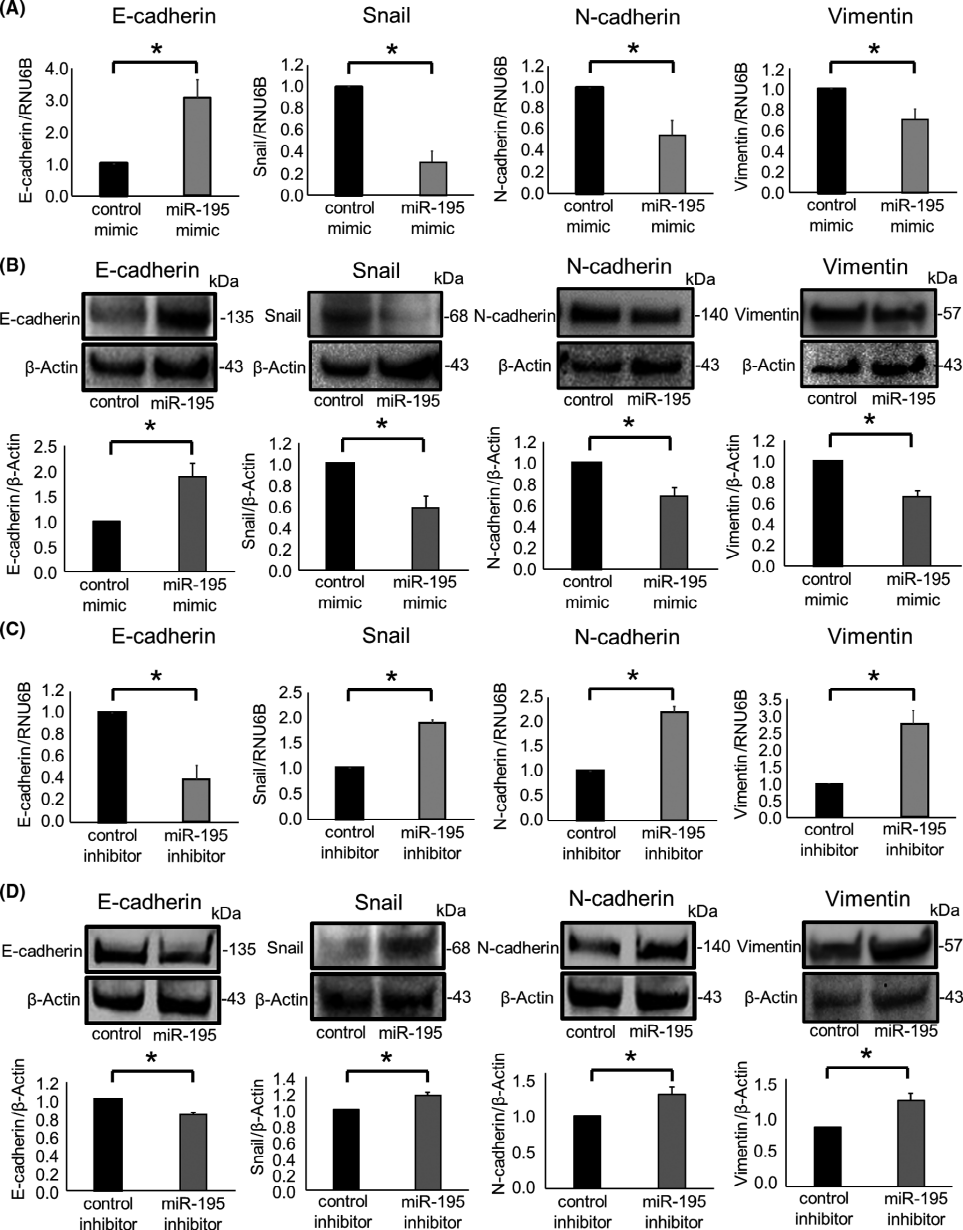
miR‐195 modulates EMT‐related genes. (A, B) HuCCT1 cells were transfected with 12.5 nm miR‐195 or a control mimic. After 48 h, RNA was isolated and analyzed by qRT‐PCR, and protein was extracted and assessed by western blot for E‐cadherin, Snail, N‐cadherin, and vimentin after 72 h. (C, D) HuCCT1 cells were transfected with 25 nm miR‐195 inhibitor or nontargeting control inhibitor. After 48 h, RNA was isolated and analyzed by qRT‐PCR, and protein was extracted and assessed by western blot for E‐cadherin, Snail, N‐cadherin, and vimentin after 72 h. A representative immunoblot is shown, along with quantitative data obtained by densitometry. Data were analyzed using Student’s *t*‐test, and bars represent mean ± SEM of three separate determinations. *P < 0.05.

miR‐195 was predicted to target the 3′UTR of Snail, an EMT‐inducible transcription factor, by bioinformatics analysis using miRNA.org (Fig. 5C). In order to validate whether Snail can be targeted by miR‐195, we performed a luciferase reporter assay using a vector encoding the Snail 3′UTR. A significant reduction in luciferase activity was observed when miR‐195 mimic and the firefly luciferase reporter vector were co‐transfected into CCA cells, suggesting that the Snail 3′UTR contains miR‐195 binding sites (Fig. 5D). Next, we modulated the expression of miR‐195 by the use of a miRNA mimic to overexpress miR‐195, or using a miRNA inhibitor to attenuate miR‐195 activity and assessed the impact on EMT in CCA cells. Compared with controls, enforced expression of miR‐195 resulted in an increase in E‐cadherin expression and concomitant reductions in Snail, N‐cadherin, and vimentin expression at both RNA and protein levels (Fig. 7A,B). In contrast, inhibiting miR‐195 reduced E‐cadherin expression and increased Snail, N‐cadherin, and vimentin expression (Fig. 7C,D). Incubation with CM derived from miR‐195‐overexpressing MMNK‐1 cells significantly increased the expression of E‐cadherin and decreased the expression of Snail, N‐cadherin, and vimentin in recipient HuCCT1 cells (Fig. 6C,D). These results indicate that validated Snail as a target of miR‐195 and show that miR‐195 could directly target Snail to suppress EMT.

### miR‐195‐enriched secretome suppresses tumor growth of CCA *in vivo*

**Fig. 8:**
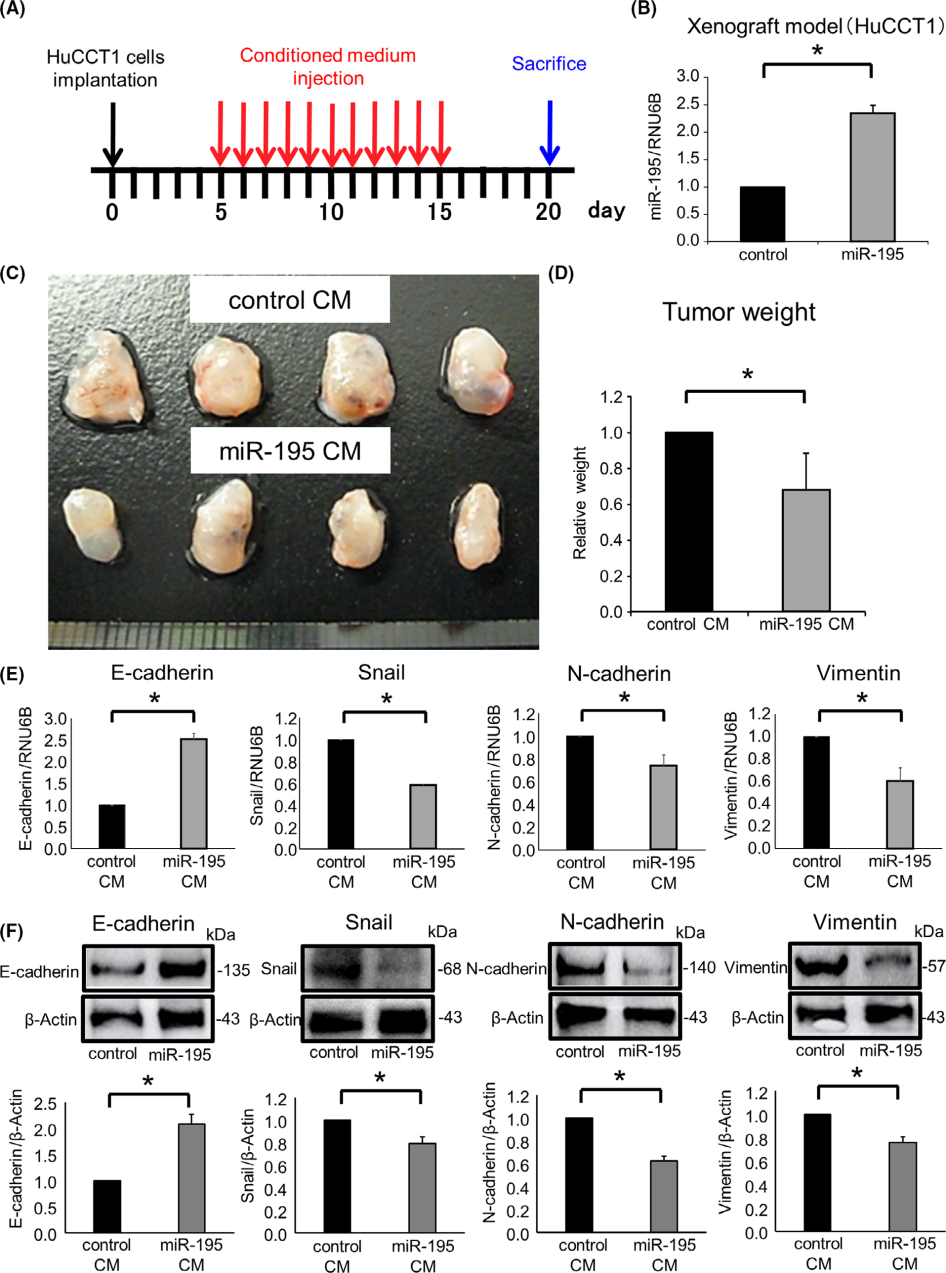
miR‐195‐enriched secretome suppresses tumor growth of CCA *in vivo*. Nude mice were injected subcutaneously into either flank with HuCCT1 cells transfected with miR‐195 or control mimic. (A) Experimental design. (B–F) The xenograft tumors were excised 20 days after the subcutaneous injections. RNA and protein were extracted from xenograft tumors. (B) MiR‐195 expression was analyzed by qRT‐PCR. (C) Excised xenograft tumors. (D) Xenograft tumor weight was analyzed. Bars represent mean ± SEM of tumor weight evaluations from four mice/group. (E) E‐cadherin, Snail, N‐cadherin, and vimentin were analyzed by qRT‐PCR. (F) E‐cadherin, Snail, N‐cadherin, and vimentin were assessed by western blot. A representative immunoblot is shown, along with quantitative data obtained by densitometry. Data were analyzed using Student’s *t*‐test, and bars represent mean ± SEM of three separate determinations. *P < 0.05.

To examine the *in vivo* impact of miR‐195 within the TME, we evaluated the effects of exogenous administration of mir‐195 enriched secretome. These studies explored the potential of miR‐195 transfer from nonmalignant cells to modulate CCA cell growth *in vivo*. HuCCT1 xenografts were established using nude mice by subcutaneous implantation. After five days, CM derived from miR‐195‐overexpressing or control MMNK‐1 cells was injected into tumor cell xenografts daily for 10 consecutive days. The mice were sacrificed, and the xenograft tumors were excised on day 20 (Fig. 8A). The transfer of miR‐195 by the systemic administration regimen was confirmed in tumor tissues (Fig. 8B). Exogenous administration of miR‐195‐enriched secretome inhibited xenograft CCA growth and reduced tumor weight (Fig. 8C,D). Moreover, a decrease in Snail, N‐cadherin, and vimentin and an increase in E‐cadherin mRNA and protein levels were observed with this treatment (Fig. 8E,F). These results suggest that the transfer of miR‐195 from normal to transformed cholangiocytes could suppress tumor cell invasion and migration *in vitro* and the growth of CCA *in vivo*, through a mechanism involving the inhibition of EMT by directly targeting Snail that is sensitive to the presence of profibrogenic TGF‐β.

## Discussion

The contributions of the tumoral and stromal cell secretome within the TME are well recognized to influence cancer growth and therapeutic responses. Our findings indicate that nonmalignant cell secretome can also contribute to the local milieu to modulate tumor cell phenotype and tumor growth. A component of this contribution in CCA is EV miR‐195 released by normal cholangiocytes. miR‐195 is an onco‐suppressive miRNA that is responsive to TGF‐β and can contribute to the growth of transformed malignant cholangiocytes through modulation of EMT. EVs from nonmalignant epithelial cells have the capacity to suppress the development of cancer [15]. However, these data indicate that they could also promote tumor growth within a profibrogenic environment that is characterized by the presence of TGF‐β and thereby emphasize a critical contribution of the microenvironmental milieu to cellular interactions between normal and transformed cells.

The association of miR‐195 with regulation of tumor growth in human CCA has several clinical and translational implications [16, 17]. A lower expression of miR‐195 in serum is related to the clinical stage and worse survival in patients with CCA [16]. Li *et al*. [17] reported that systemic injections of miR‐195‐loaded stroma‐derived EVs could suppress tumor growth and prolong survival in a rat model of CCA. Importantly, miR‑195 may act as a tumor suppressor gene in other cancers as well, and reduced expression of miR‐195 has been observed in hepatocellular carcinoma (HCC) [18, 19, 20], breast cancer [21, 22], prostate cancer [23, 24], gastric cancer [25], and colon cancer [26]. Further exploration of TME‐driven effects on nontransformed cell effects on tumor growth in these settings could provide further mechanistic insights into tumor growth as well as identify potential therapeutic approaches. Other groups have shown that miR‐195 can regulate TGF‐β‐related signaling to modulate tumor growth or fibrosis [27, 28]. Although the mechanisms by which TGF‐β can suppress miR‐195 expression have not been described in these studies or examined in the current study, they warrant further evaluation.

Defining the detailed mechanism by which miR‐195 exerts an onco‐suppressive effect will be important to understand its potential as therapeutic target. We show that EV‐encapsulated miR‐195 can regulate the EMT by directly targeting Snail. Exploration of the miR‐195‐Snail pathway and its downstream impact will yield further insights into specific contributions of EV‐RNA transfer on mechanisms underlying tumor invasion and metastasis. Interestingly, miR‐195 has been reported to inhibit metastasis by regulating the EMT and targeting the Yes‐associated protein (YAP) in HCC [20] and can inhibit the EMT by targeting fibroblast growth factor (FGF) 2 in prostate cancer [23]. Both YAP and FGF receptor signaling are important in cholangiocarcinoma growth [29, 30]. Thus, other targets of miR‐195 on cancer‐related pathways or EMT‐driving processes or effects are possible although these were not specifically in the present study.

These data emphasize the central role of the cellular secretome within the TME and the possibility that secretome products other than EVs and their contents, such as extracellular RNA within RNA complexes or lipoproteins, could potentially contribute to the observed differences. Rather than EVs, we focused on the use of CM to more closely recapitulate the secretome from donor nonmalignant cholangiocytes for our *in vivo* studies. This scenario more closely recapitulates the *in vivo* situation, in which multiple factors may act in concert to impact on an outcome driven by important contributing drivers. Through the demonstration that systemic administration of miR‐195‐enriched CM suppressed EMT and the growth of xenografted tumors, these studies highlight the important contribution of miR‐195 and further justify efforts to modulate EV‐mediated miR‐195 as a new therapeutic target for human CCA.

EV‐mediated transfer of ncRNA can contribute to the initiation, invasion, metastasis, and recurrence of cancer through modulation of signaling pathways in tumor cells [7], or through effects on other cells within the local TME or even within distant tissues [15, 31]. These responses are likely to be tumor‐specific. Similar to our observations with miR‐195, different onco‐suppressive miRNA such as miR‐143 have been reported in other tumors. Expression of miR‐143 is higher in normal as compared with malignant prostate cells. Moreover, EVs derived from normal prostate cells can transfer miR‐143 as a growth‐inhibitory signal to cancerous cells, both *in vitro* and *in vivo* [32, 33]. Of note, functional variants in the miR‐143 promoter contributed to prostate cancer risk in a Chinese population [34]. Demonstration of the contributory roles of EV‐mediated RNA within defined tumor and tissue contexts could further lead to new mechanistic insights into tumor growth.

## Conclusion

In this study, we evaluated the impact of EV RNA signaling by normal cholangiocytes and identified miR‐195 as a suppressor of tumor cell invasion and migration and its effects on EMT processes. These observations not only provide mechanistic insights into intercellular communication through EV RNA transfer within the TME to modulate tumor growth and spread, but they also emphasize potential strategies to suppress tumor growth in CCA through the application of EV‐based nanotherapeutics.

## Conflict of interest

The authors declare no conflict of interest.

## Author contributions

YO and KT conceived and designed the project. YO performed the experiments and analyzed the data. YO and KT wrote the manuscript. SO, YT, MO, IY, and KA reviewed the data. SF and MH conducted experiments and data analysis. TP provided critical revisions. All authors reviewed and approved the final manuscript.

## Data Availability

The experimental data generated and analyzed during this study are included in this published article and are available from the corresponding author on reasonable request. In addition, miRNA microarray data discussed in this publication are accessible through GEO Series accession numbers GSE104629 and GSE121512.
